# Geriatric Trauma: Identifying Research Gaps and Opportunities for Improvement

**DOI:** 10.7759/cureus.65135

**Published:** 2024-07-22

**Authors:** Tawfeeq Altherwi

**Affiliations:** 1 Emergency Medicine, Faculty of Medicine, Jazan University, Jazan, SAU

**Keywords:** trending topics, hotspots, conceptual mapping, bibliometrics, geriatric trauma

## Abstract

Geriatric trauma is a critical area of research owing to the increasing elderly population and the unique challenges associated with their injuries. This study aimed to explore the intellectual structure, growth, spatial analysis, seminal publications, most frequent keywords, trending topics, conceptual structure, and thematic evolution of geriatric trauma research (GTR). A comprehensive analysis was conducted using VOSviewer (Centre for Science and Technology Studies, Leiden University, The Netherlands) and Biblioshiny (K-Synth Srl, University of Naples Federico II, Italy) to examine a corpus of scholarly publications (N = 682) related to GTR (2004-2024). Bibliographic data were collected from the Scopus database and analyzed to highlight the key findings and trends. The analysis revealed the leading contributors to GTR. Over the years, there has been an increased interest in geriatric trauma, as demonstrated by the increasing trend in research publications. Collaboration patterns among nations were determined through spatial analysis, and insights into significant topics and their influence were offered by influential publications. Keywords used frequently as well as current issues formed part of this study’s findings, which give insight into what was most focused on within the GTR. Themes and their development over time were made explicit by revealing their conceptual structures and thematic evolutions. GTR has increased significantly. Interdisciplinary approaches are suggested for future research. Furthermore, gaps need to be addressed using technological advancements that will help improve geriatric trauma management and lead to better patient outcomes.

## Introduction and background

Geriatric trauma is a major public health concern involving injuries sustained by older adults, typically aged 65 years and above, due to accidents, falls, or other traumatic incidents [1,2]. This vulnerable population experiences age-related physiological changes, comorbidities, and reduced functional reserves, making them more susceptible to injury. Older adults suffer from various forms of chronic diseases, such as high blood pressure or type 2 diabetes, which are common among the elderly, making it easy for them to fall victim when accidents occur [3,4].

Research has demonstrated that geriatric trauma contributes substantially to hospitalization and mortality among older adults, with falls being a leading cause of fractures, head injuries, and other severe complications. Specific risk factors, such as polypharmacy, cognitive impairment, and environmental hazards, further contribute to the occurrence and severity of geriatric trauma [4,5]. Geriatric injury is one of the primary reasons for hospital admissions among the elderly. The major cause of fracture, which typically results from falling for elderly people in hospitals, is fall-related injuries that mostly originate from falling [1].

The global demographic shift towards an aging population, projected to reach two billion people aged 60 years and older by 2050, underscores the urgency of addressing geriatric trauma. A comprehensive understanding of this issue is essential for developing effective prevention strategies, optimizing trauma care systems, and improving patient outcomes [6,7]. Epidemiological research has provided valuable insights into the impact of geriatric trauma [4,8,9]. Older adults experiencing trauma encounter unique challenges during the recovery process, complicated by preexisting comorbidities that increase the risk of complications. The significant healthcare burden associated with geriatric trauma necessitates longer hospital stays than younger individuals need rehabilitation services must be performed so as to restrict functional decline rate together with a multi-disciplinary approach [10]. Age-friendly environments can prevent these accidents from happening [11].

Although numerous studies have been conducted regarding old persons’ accidents, there are still knowledge gaps regarding various aspects related to this issue, which need further investigation [3,4,8,12]. Prevention strategies, rehabilitation approaches, and optimized healthcare interventions tailored to this population require investigation [11]. It is important to know the long-term outcomes and quality of life after geriatric trauma; hence, specific programs are needed for interventions in this area [13]. A bibliometric study on geriatric trauma will help to identify research gaps in this area, provide insights into the existing literature, and guide future studies.

This study aimed to examine the thematic evolution and conceptual structure of geriatric trauma research (GTR) based on the identified trending topics, most frequent keywords, seminal GTR works, spatial analysis of collaborative and impactful GTR, growth patterns, and intellectual structure. This study aimed to provide insights into the research landscape, identify key research areas, influential works, collaboration patterns, and the overall development of knowledge in the field of geriatric trauma.

## Review

Materials and methods 

Database Selection and Data Mining

This study opted for the Scopus database because it has numerous advantages over the Web of Science (WOS), Google Scholar, and PubMed [14]. The Medical Subject Heading (MeSH) database was used to construct GTR-related search terms [15]. A search query was created using keywords such as “Geriatric trauma,” “Geriatric injury,” “Elderly AND trauma,” and “Aged AND injury.” Appropriate operators (OR, AND) were used to combine them. The first search yielded 1,089 documents. To refine the results, additional criteria were applied, including restrictions on publication year (2004-2024) and language (English). In total, 682 GTR-related documents were found. The use of original articles and the English language was justified in this study to ensure robustness, accessibility, comparability, global relevance, and efficient resource utilization. However, language and publication biases should also be considered.

Quality Control

To ensure the accuracy of the chosen bibliographic data, each document was subjected to a screening process. This involved reviewing all titled abstracts along with keywords contained within them so as not to allow any irrelevant materials into this investigation; a simple venture considering that they are all about geriatric patients who have been injured physically or mentally during their lifetime. The policy aimed to encompass only publications that focused directly on geriatric trauma. This procedure was performed on the top-cited documents related to GTR.

Data Analysis

Two applications, Biblioshiny and VOSviewer [16,17], were used for the bibliometric analysis in this article. The function of these applications is mainly to help in the drawing of visualizations and statistical analyses on a web-based platform that will facilitate the visualization and statistical analysis of bibliographic data. These applications were specifically chosen, among others. This enabled us to study how geriatric trauma appears from the viewpoint of bibliometrics by analyzing it from different angles.

Data Visualization

Bibliometrics were visualized using VOSviewer and Biblioshiny software. For instance, graphs have been produced, such as co-authorship networks or keyword co-occurrences. On the other hand, Biblioshiny offers several interactive visualizations, such as bar charts, scatter plots, and word clouds depicting different aspects of bibliometric indicators and trends. The total number of articles published by authors through international collaboration is known as total link strength (TLS). That is, the country with the highest level of collaboration typically exhibits high values for total link strength. Research themes within a field can be visualized in a two-dimensional strategic diagram based on their Callon's centrality and density measures. The diagram classifies themes into four groups: motor themes (upper-right quadrant) are well-developed and vital for the field, specialized and peripheral themes (upper-left quadrant) have strong internal ties but limited external importance, weakly developed and marginal themes (lower-left quadrant) represent emerging or disappearing areas, and important but underdeveloped themes (lower-right quadrant) encompass transversal and general basic topics. This diagram provides a concise overview of the research field's structure and theme characteristics [16,17].

Results

Intellectual Structure

The intellectual structure of the GTR reveals the most productive authors, affiliations, countries/territories, and source titles. Joseph, B. and Konda, S.R. are the most prolific authors with 29 publications each, followed by Egol, K.A. with 27 publications, Knobe, M. with 20 publications, and Pape, H.C. with 15 publications. The University of Arizona and the University of Arizona College of Medicine - Tucson are the top affiliations, both with 28 publications, while the NYU (New York University) Langone Orthopedic Hospital, Jamaica Hospital Medical Center, and NYU Langone Health also have significant contributions. The United States led in GTR with 415 publications, followed by Germany with 74 publications, Switzerland with 44 publications, the United Kingdom with 25 publications, and the Netherlands with 20 publications. Geriatric Orthopaedic Surgery and Rehabilitation is the most prominent source title, with 101 publications, followed by American Surgeon, Injury, Journal of Trauma and Acute Care Surgery, and Journal of Surgical Research.

Growth

**Figure 1:**
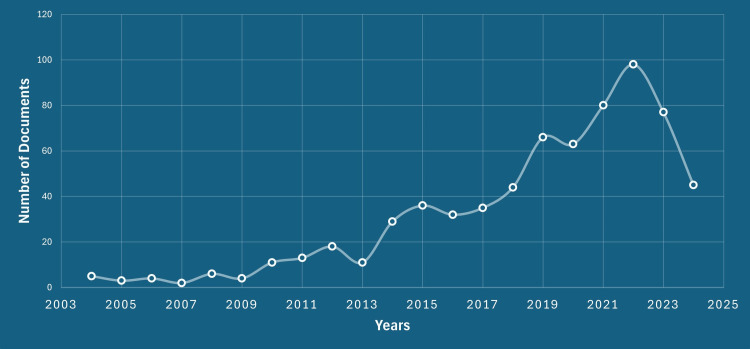
Annual growth (2004-2024) Y-axis: Number of articles published; X-axis: Years

The growth of geriatric trauma research is depicted in Figure 1, showing the number of publications and percentage distribution across different years. By 2024, there were 45 publications, accounting for 6.60% of the total. The year 2022 had the highest number of publications, followed by 2023. In 2021, there were 80 publications (11.73%), whereas 2020 had 63 publications (9.24%). 

Spatial Analysis of Collaborative and Impactful GTR

**Figure 2:**
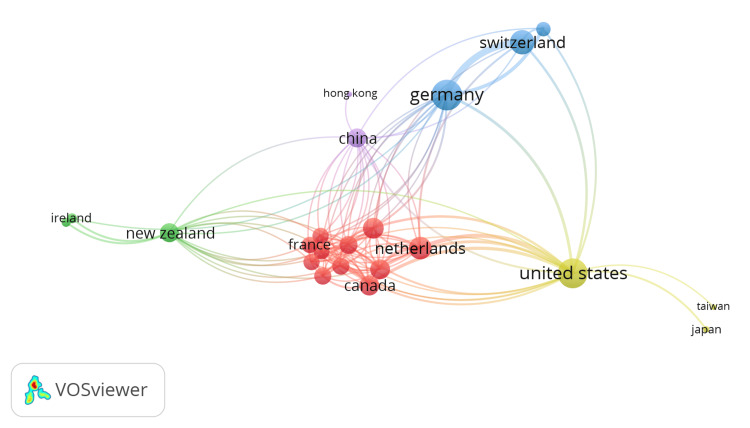
Spatial analysis of collaborative GTR. The size of each node corresponds to the TLS value of the respective country, indicating the level of collaboration between researchers from that country and others. Larger nodes indicate higher TLS values, reflecting stronger collaborative efforts. GTR: geriatric trauma research; TLS: total link strength

In the GTR, Germany and the United States exhibited the highest TLS, indicating strong collaboration among researchers (Figure 2). Germany has a TLS of 46, whereas the United States follows closely with 44. Switzerland ranks third, with 30, and the Netherlands and Australia have TLS values of 26 and 21, respectively. The nodes in Figure 2 represent the TLS for each country. In terms of citations, the United States leads with 7615, followed by Germany with 626, Canada with 439, Switzerland with 321, Austria with 238, and the United Kingdom with 225. These countries have made significant contributions to the field and received notable recognition in terms of collaborative research and citations.

Seminal GTR

Seminal publications in GTR cover a variety of topics related to the evaluation, management, and outcomes of elderly trauma patients (Table 1). These topics include age cutoffs for increased mortality in elderly trauma patients, the reliability of presenting vital signs in geriatric blunt trauma patients, predicting hospital discharge disposition in geriatric trauma patients, the role of frailty in predicting outcomes among geriatric trauma patients, validating the trauma-specific frailty index for geriatric trauma patients, the impact of advanced age on trauma triage decisions and outcomes, mortality of patients treated in a hip fracture program for elders, contemporary patterns of trauma deaths with an emphasis on older individuals, and the circadian rhythm of salivary cortisol in Holocaust survivors with and without post-traumatic stress disorder (PTSD). The document “Superiority of frailty over age in predicting outcomes among geriatric trauma patients: A prospective analysis” [9] scored 361 citations and 32.82 as the citation average. These seminal publications have significantly contributed to our understanding of geriatric trauma, shaping clinical practice and research in this field.

**Table 1:** Highly cited geriatric trauma research CA: citation average

Title	Source	Year	Citations	CA
“Superiority of frailty over age in predicting outcomes among geriatric trauma patients: a prospective analysis” [9]	JAMA Surgery	2014	361	32.82
“Validating trauma-specific frailty index for geriatric trauma patients: a prospective analysis” [12]	Journal of the American College of Surgeons	2014	217	19.73
“Epidemiology and contemporary patterns of trauma deaths: changing place, similar pace, older face” [11]	World Journal of Surgery	2007	176	9.78
“Evaluation and management of geriatric trauma: an Eastern Association for the Surgery of Trauma Practice Management guideline” [6]	Journal of Trauma and Acute Care Surgery	2012	172	13.23
“The impact of advanced age on trauma triage decisions and outcomes: a statewide analysis” [10]	American Journal of Surgery	2009	155	9.69
“Circadian rhythm of salivary cortisol in Holocaust survivors with and without PTSD” [7]	American Journal of Psychiatry	2005	149	7.45
“Normal presenting vital signs are unreliable in geriatric blunt trauma victims” [1]	Journal of Trauma - Injury, Infection and Critical Care	2010	139	9.27
“Predicting hospital discharge disposition in geriatric trauma patients:Is frailty the answer?” [2]	Journal of Trauma and Acute Care Surgery	2014	130	11.82
“Identification of an age cutoff for increased mortality in patients with elderly trauma” [18]	American Journal of Emergency Medicine	2010	128	8.53
“The 1-Year Mortality of Patients Treated in a Hip Fracture Program for Elders” [19]	Geriatric Orthopaedic Surgery & Rehabilitation	2010	308	20.53

Most Frequent Keywords

**Figure 3:**
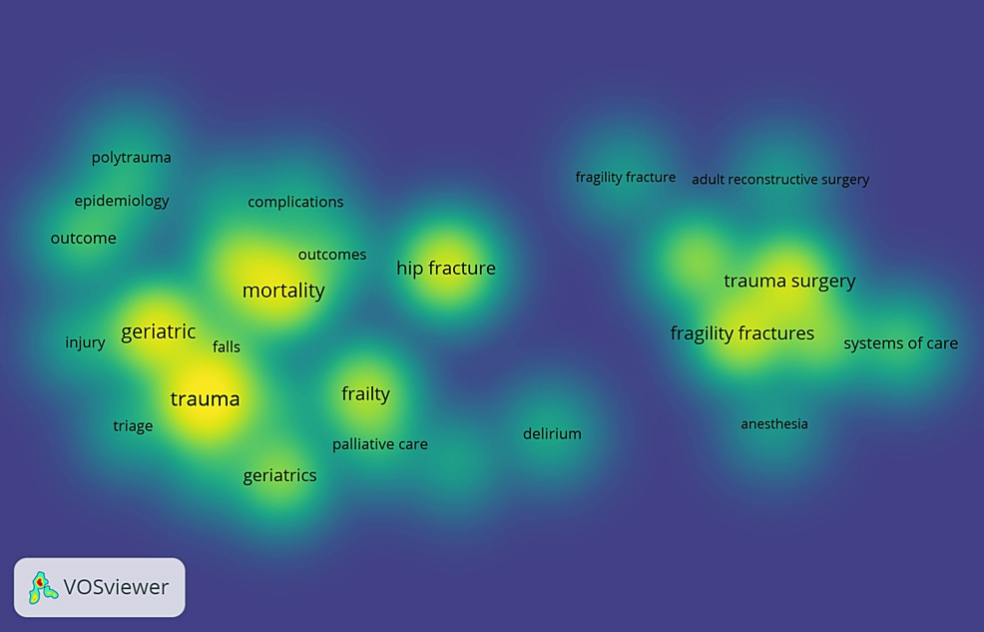
Most frequent author’s keywords. Co-occurrence was analyzed using VOSviewer. The darker shades of yellow indicate higher frequency, while lighter shades represent lower frequency. By observing the color variations in the heatmap, one can quickly discern the relative prominence of keywords based on their frequency.

The most recurrent author keywords in the GTR indicate prominent areas of focus within the field. The top keywords and their occurrences (Figure 3) are as follows: "geriatric trauma" (n=538), "mortality" (n=69), "hip fracture" (n=67), "trauma surgery" (n=64), "fragility fractures" (n=62), "frailty" (n=51), "elderly" (n=47), "osteoporosis" (n=41), "geriatrics" (n=40), "geriatric medicine" (n=36), "outcome" (n=23), "systems of care" (n=23), "falls" (n=19), "outcomes" (n=18), "anticoagulation" (n=16), "palliative care" (n=15), and "delirium" (n=14).

Trending Topics

**Figure 4:**
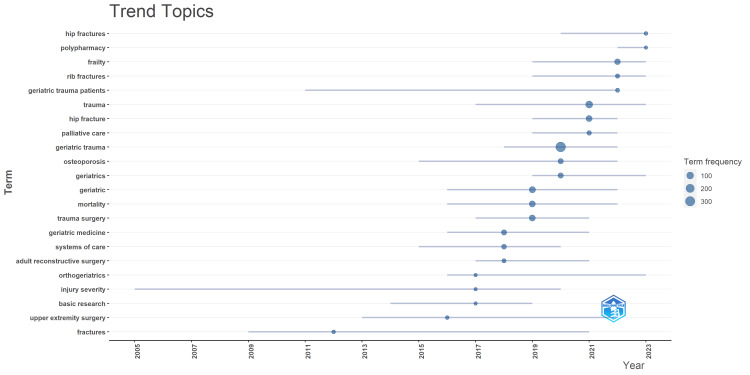
Trends in the GTR over the last 20 years. The diagram depicts the research topic's time length, with lines indicating the period and blue circles representing the occurrence of the term. This figure was produced using Biblioshiny and BibTex data files. GTR: geriatric trauma research

Based on the occurrence of the author's keywords (Figure 4), the trending topics in GTR are “polypharmacy,” “hip fractures,” “geriatrics,” “palliative care,” “frailty,” and “rib fractures.” The lines in Figure 4 represent the shelf life of the trending topic, and the circles indicate the frequency.

Conceptual structure

**Figure 5:**
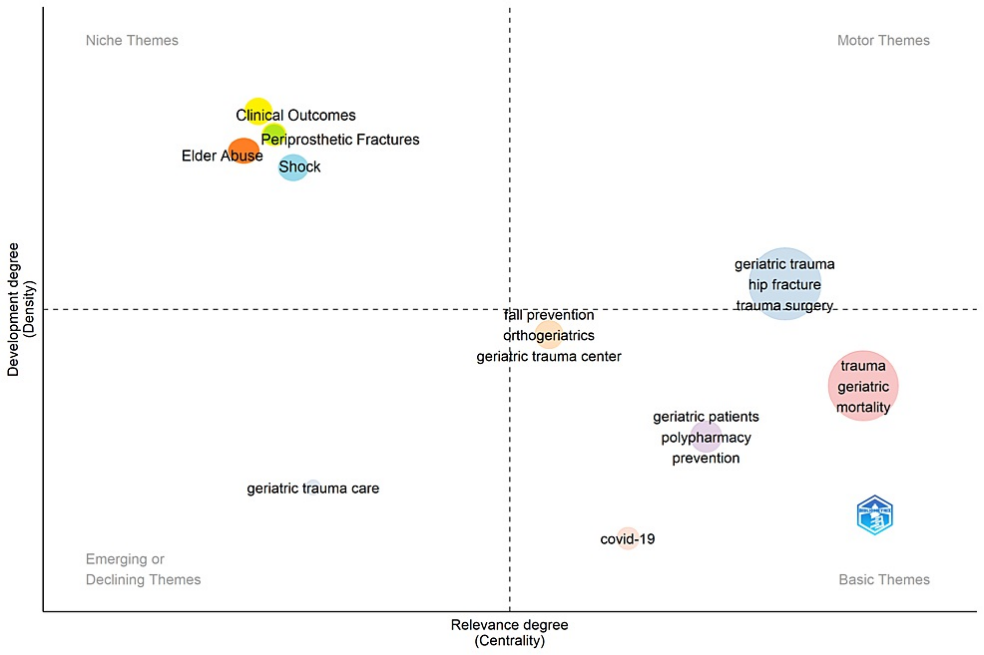
Thematic map of the GTR. Four quadrants that stand for the significance and evolution of research topics are formed from thematic maps according to centrality and density. This figure was produced with the BibTex data file and the Biblioshiny program. GTR: geriatric trauma research

The thematic map of the GTR reveals 10 clusters: trauma, geriatric trauma, geriatric patients, fall prevention, shock, elder abuse, COVID-19, geriatric trauma care, clinical outcomes, and periprosthetic fractures. These clusters represent important areas of research in the field, focusing on understanding and addressing the unique aspects of trauma in the elderly, improving care and outcomes, and exploring related topics, such as fall prevention, elder abuse, and the impact of COVID-19. A thematic map of GTR is shown in Figure 5 and Table 2.

**Table 2:** Classifications of the clusters of GTR. GTR: geriatric trauma research

Cluster	Callon Centrality	Callon Density	Classification	Major terms
Trauma	2.41	29.12	Basic	Trauma, geriatric, mortality
Geriatric Trauma	1.85	32.16	Motor	Geriatric trauma, hip fracture, trauma surgery
Geriatric Patients	0.67	28.45	Basic	Geriatric patients, polypharmacy, prevention
Fall Prevention	0.05	31.00	Basic	Fall prevention, orthogeriatrics, geriatric trauma center
Shock	0.00	33.33	Niche	Shock
Elder Abuse	0.00	33.33	Basic	Elder abuse
COVID-19	0.11	12.50	Basic	COVID-19
Geriatric Trauma Care	0.00	25.00	Emerging	Geriatric trauma care
Clinical Outcomes	0.00	33.33	Niche	Clinical outcomes
Periprosthetic Fractures	0.00	33.33	Niche	Periprosthetic fractures

Thematic evolution

**Figure 6:**
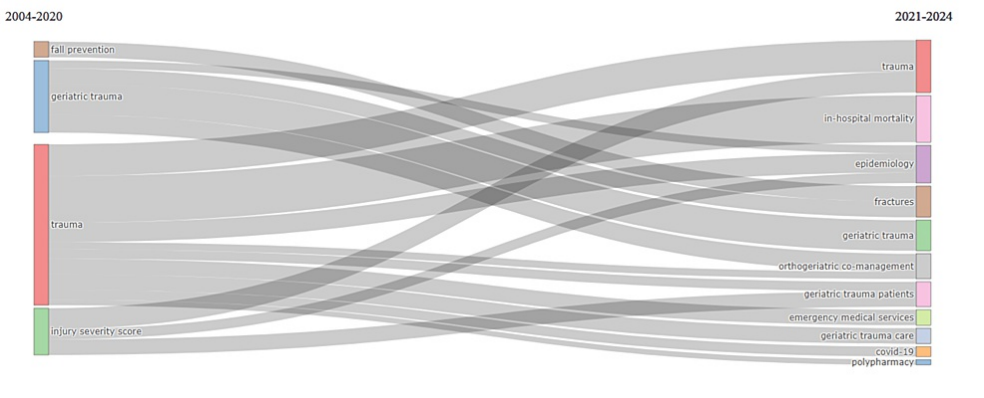
Thematic evolution of the GTR. 2020 was an important year in the evolution of the major subjects. Throughout the study period, the research scope expanded to encompass COVID-19, emergency medical services, and trauma epidemiology. However, despite these additions, the areas of geriatric trauma and trauma remained consistently prominent research subjects. This figure was produced with the BibTex data file and the Biblioshiny program. GTR: geriatric trauma research

Figure 6 illustrates the thematic evolution of the GTR. It shows a transition from fall prevention to fractures, a shift from geriatric trauma to epidemiology, and the emergence of new topics, such as orthogeriatric co-management and polypharmacy. The research focus expanded to include COVID-19, emergency medical services, and trauma epidemiology. Despite these changes, geriatric trauma and trauma remained consistent research areas throughout the study period.

Discussion

This study aimed to analyze the thematic evolution and conceptual structure of GTR based on the identified trending topics, most frequent keywords, seminal GTR works, spatial analysis of collaborative and impactful GTR, growth patterns, and intellectual structure. This study aimed to provide insights into the research landscape, identify key research areas, influential works, collaboration patterns, and the overall development of knowledge in the field of geriatric trauma.

Joseph, B., from the Department of Surgery, College of Medicine, University of Arizona, USA, stands out as the most prolific scholar in GTR. His notable contributions include studies on various aspects of geriatric trauma, such as the impact of age on falls from ladders, the role of frailty in predicting discharge disposition and outcomes, the validation of a trauma-specific frailty index, the influence of frailty on failure-to-rescue rates, the relationship between upper-extremity function and health outcomes in older adults hospitalized for falls, mortality rates after trauma laparotomy in geriatric patients, the association between sarcopenia and frailty, the effect of frailty on the acute inflammatory response after trauma, and the use of the shock index to predict mortality in geriatric trauma patients [2-5,8,9,12,20-22]. Joseph's extensive research portfolio significantly contributes to the understanding and advancement of geriatric trauma care.

The United States’ leadership in GTR and scientific advancements have a positive impact on the quality of life of older people through advanced healthcare [23,24]. Factors such as research financing, strong institutions, teamwork, technology progressions, academic liberty, and intellectual property rights [25] account for the leading status of the United States with respect to GTR. These scientific milestones result in biomedical research, innovations in healthcare access and delivery systems, advancing aging-related diseases, influence on healthcare policy, and promotion of longevity and healthy aging [26]. This study corroborates prior bibliometric studies that found that the United States is a leader in the geriatric research area [23,27-30].

Figure 6 shows the dynamic nature of the GTR over time. Several shifting themes also highlight changes in priorities within an increasing field of interest in geriatrics. Changing from fall prevention to fractures, moving from geriatric trauma to epidemiology, and the emergence of new topics such as orthogeriatric co-management [31] and polypharmacy [32,33] signify changing priorities and emerging areas of interest within geriatric research. The inclusion of COVID-19, emergency medical services, and trauma epidemiology reflects the field's response to current global health challenges [34]. Despite these changes, geriatric trauma and trauma remain consistent research areas, highlighting their significance. This evolution shows a growing understanding of the complexities of geriatric trauma care and a commitment to improving the outcomes and quality of care for older adults experiencing trauma.

Table 2 presents the classifications of the clusters in the GTR. The "Trauma" cluster stands out with high centrality (2.41) and density (29.12), focusing on trauma, geriatric issues, and mortality. "Geriatric Trauma" explores specific aspects such as hip fractures and trauma surgery. "Geriatric Patients" examines polypharmacy and prevention, while "Fall Prevention" emphasizes strategies to reduce fall-related injuries. Other clusters include "Shock," "Elder Abuse," "COVID-19," "Geriatric Trauma Care," "Clinical Outcomes," and "Periprosthetic Fractures," each with its niche focus. Table 2 demonstrates the diverse research landscape within geriatric trauma, showcasing broad and specialized investigation areas. The diversity of GTR is consistent with previous bibliometric studies that conceptualized research structures in the elderly [23,30].

Polypharmacy, the use of multiple medications by an individual [32], has emerged as a trending topic in GTR. With the aging population and increasing prevalence of chronic diseases among older adults, the need to manage multiple health conditions has led to a higher incidence of polypharmacy [33,35]. This phenomenon has raised concerns owing to its association with adverse outcomes in geriatric trauma patients, including medication interactions, side effects, errors, and non-adherence. Additionally, age-related changes in drug response and metabolism in older adults further amplify the risks associated with polypharmacy [32,33,35-37]. In response, GTR researchers are actively studying the prevalence, patterns, and consequences of polypharmacy to develop interventions that optimize medication use, enhance safety, and minimize risks for geriatric trauma patients, ultimately aiming to improve their outcomes and quality of care. The current findings are in line with previous research on the emergence of polypharmacy in the elderly [32,33,35,36].

To overcome the limitations of using a single database source and focusing on English literature, researchers can expand data sources by including additional databases, employing a multilingual search strategy, collaborating with subject experts, conducting a systematic review, and considering incorporating grey literature.

## Conclusions

Mapping of GTR reveals essential insights. The field has experienced substantial growth, with notable contributions from authors and affiliations in the United States, Germany, and Switzerland. The identified research gaps include limited studies on specific topics such as polypharmacy, palliative care, and delirium in geriatric trauma, as well as a need for more comprehensive investigations into the long-term outcomes and quality of life of elderly trauma patients. Future recommendations include fostering international collaborations to advance GTR, promoting interdisciplinary approaches to address the multifaceted needs of elderly patients, and leveraging emerging technologies and data analytics to enhance risk assessment, personalized interventions, and post-discharge care. Additionally, there is a need for more studies focusing on the impact of social determinants of health, disparities in access to care, and the effectiveness of geriatric trauma protocols in different healthcare settings. These actions can improve the understanding, prevention, and management of geriatric trauma, ultimately enhancing patient outcomes and healthcare delivery.
